# Integrated exposure–response analysis of efficacy and safety of lurbinectedin to support the dose regimen in small-cell lung cancer

**DOI:** 10.1007/s00280-021-04366-3

**Published:** 2021-11-05

**Authors:** Carlos Fernández-Teruel, Salvador Fudio, Rubin Lubomirov

**Affiliations:** Pharma Mar, S.A., Avda. De los Reyes, 1, Pol. Ind. La Mina-Norte, 28770 Colmenar Viejo, Madrid Spain

**Keywords:** Lurbinectedin, Exposure–response, Pharmacokinetics, Small-cell lung cancer, Clinical utility index

## Abstract

**Purpose:**

These exposure–response (E–R) analyses integrated lurbinectedin effects on key efficacy and safety variables in relapsed SCLC to determine the adequacy of the dose regimen of 3.2 mg/m^2^ 1-h intravenous infusion every 3 weeks (q3wk).

**Methods:**

Logistic models and Cox regression analyses were applied to correlate lurbinectedin exposure metrics (AUC_*tot*_ and AUC_*u*_) with efficacy and safety endpoints: objective response rate (ORR) and overall survival (OS) in SCLC patients (*n* = 99) treated in study B-005 with 3.2 mg/m^2^ q3wk, and incidence of grade 4 (G4) neutropenia and grade 3–4 (G ≥ 3) thrombocytopenia in a pool of cancer patients from single-agent phase I to III studies (*n* = 692) treated at a wide range of doses. A clinical utility index was used to assess the appropriateness of the selected dose.

**Results:**

Effect of lurbinectedin AUC_*u*_ on ORR best fitted to a sigmoid-maximal response (*E*_max_) logistic model, where *E*_max_ was dependent on chemotherapy-free interval (CTFI). Cox regression analysis with OS found relationships with both CTFI and AUC_*u*_. An *E*_max_ logistic model for G4 neutropenia and a linear logistic model for G ≥ 3 thrombocytopenia, which retained platelets and albumin at baseline and body surface area, best fitted to AUC_*tot*_ and AUC_*u*_. AUC_*u*_ between approximately 1000 and 1700 ng·h/L provided the best benefit/risk ratio, and the dose of 3.2 mg/m^2^ provided median AUC_*u*_ of 1400 ng·h/L, thus maximizing the proportion of patients within that lurbinectedin target exposure range.

**Conclusions:**

The relationships evidenced in this integrated E–R analysis support a favorable benefit-risk profile for lurbinectedin 3.2 mg/m^2^ q3wk.

**Trial registration:**

Clinicaltrials.gov: NCT02454972; registered May 27, 2015.

**Supplementary Information:**

The online version contains supplementary material available at 10.1007/s00280-021-04366-3.

## Introduction

Lung cancer is the leading cause of cancer deaths worldwide. Small-cell lung cancer (SCLC) accounts for about 15% of lung cancers overall and is a particularly aggressive neoplasm with a 5-year survival rate of < 5%. Little progress has been made in improving the outcome for patients with this malignancy over the past 30 years. When patients with SCLC relapse, few therapeutic options are available. Until recently, topotecan was the only approved drug for second-line treatment of patients with a chemotherapy-free interval (CTFI) longer than 60 days. However, topotecan use is challenging because of its associated hematological toxicities and relatively modest clinical benefit (response in around 16% of patients and median overall survival of 6–8 months) [1–6].

Lurbinectedin is a selective inhibitor of oncogenic transcription that binds preferentially to guanines located in the GC-rich regulatory areas of DNA gene promoters [7, 8]. The drug, thus, prevents binding of transcription factors to their recognition sequences, thereby inhibiting oncogenic transcription and leading to cell cycle arrest and tumor cell apoptosis [9]. Lurbinectedin has a direct effect on the tumor microenvironment by modifying the immune-regulatory properties of tumor associated macrophages, where it significantly inhibits the transcription of CCL2, CXCL8, and VEGF [10].

Lurbinectedin is highly protein-bound, and metabolism by cytochrome P450 (CYP) 3A is the major clearance mechanism [11]. Main metabolites of lurbinectedin did not show a significant contribution to the overall active moiety [12]. A population pharmacokinetic (PopPK) analysis [13] with data from 443 cancer patients suggested that an open three-compartment model with linear disposition described the time course of total plasma lurbinectedin concentrations. The estimate for the total body clearance of drug from plasma (CL_*tot*_) was 11.2 L/h, corresponding to a blood CL of ~ 17 L/h, thus reflecting a low extraction ratio of 0.19. Apparent volume at steady state was 438 L. High α-1-acid glycoprotein (AAG), C-reactive protein (CRP), and low albumin reduced clearance by 28%, 20%, and 20%, respectively. Co-administration of CYP3A inhibitors reduced clearance by 30%.

The first-in-human dose-finding study of lurbinectedin in patients with advanced solid tumors (A-001) [14] defined the recommended dose (RD) at 7.0 mg flat dose (FD) (equivalent to 4.0 mg/m^2^) as a 1-h infusion every 3 weeks (q3wk), since pharmacokinetic analyses showed no relationship between CL and body surface area (BSA). Mild and moderate reversible myelosuppression was the most relevant toxicity. A second dose-finding study (A-005) [15] exploring an alternative regimen (days 1 and 8 q3wk) found a higher incidence of dose-limiting toxicities (21% vs. 7%) at the RD (5 mg FD), more cycle delays, and poorer treatment compliance than the A-001 study. After using the 7.0 mg FD q3wk regimen in early phase II studies, semi-mechanistic pharmacokinetic-pharmacodynamic analyses were developed to model the time course of neutrophils and platelets and to explore alternative dose regimes [16]. Simulations showed that BSA-based dosed 3.2 mg/m^2^ q3wk reduced the incidence of G4 neutropenia and thrombocytopenia by 20% and 80%, respectively, compared to 7.0 mg FD q3wk. Therefore, 3.2 mg/m^2^ q3wk was selected as the starting dose for further studies.

Clinical efficacy and safety of lurbinectedin in patients with relapsed SCLC (*n* = 105) as well as other aggressive malignancies (*n* = 230) were evaluated in phase II, single-arm basket study B-005 [17]. Patients received lurbinectedin 3.2 mg/m^2^ q3wk, with support of secondary prophylaxis with granulocyte colony-stimulating factor (G-CSF) when needed. In the overall population (*n* = 335), the incidences of G4 neutropenia (< 0.5 × 10^9^/L) and G ≥ 3 thrombocytopenia (< 50 × 10^9^/L) in cycle 1 were 16.2% and 6.0%, respectively. In the SCLC cohort, lurbinectedin was efficacious in increasing the median objective response rate (ORR) in patients with resistant (22% [95% CI 11.2–37.1] and sensitive disease 45.0% [95% CI 32.1–58.4], by Independent Review Committee [IRC]) (*n* = 96), and in extending the overall survival (OS) in patients with resistant (5.0 months [95% CI 4.1–6.3]) and sensitive disease (11.9 months [95% CI 9.7–16.2]). In the responders, median duration of response was 5.1 months (95% CI 4.9–6.4 months). Based on these results, on June 15, 2020, the US Food and Drug Administration (FDA) granted accelerated approval to lurbinectedin monotherapy in SCLC that has relapsed from platinum compound-based first-line chemotherapy [11].

For the new drug application, the FDA requested justification of the 3.2 mg/m^2^ q3wk dose regimen with integrated exposure–response (E–R) analysis for efficacy and safety, which is presented herein.

Therefore, the purpose of this study was to characterize the relationships between lurbinectedin plasma exposure and both efficacy and safety endpoints, and to evaluate the potential influences of risk factors associated with these outcomes. A clinical utility index (CUI) was developed to manage safety and efficacy as a single measure, with the aim of determining the adequacy of the selected dose regimen.

## Methods

### Patients

The database from a previous PopPK analysis [13] was updated with late phase II and III studies and restricted to patients given lurbinectedin as a single agent (*n* = 755), as summarized in Online Resource Supplementary Table S1. Exposure-efficacy analyses were based on available exposure and efficacy data from SCLC patients treated with single-agent lurbinectedin from study B-005 (*n* = 99), and exposure-safety analyses were based on all single-agent lurbinectedin studies in non-hematological malignancies when the agent was given q3wk (*n* = 644) (see Online Resource Supplementary Table S1). All studies were carried out in accordance with principles for human experimentation as defined in the Declaration of Helsinki and were approved by the human investigational review board/ethics committee of each trial center, as required by International Council for Harmonisation of Technical Requirements for Pharmaceuticals for Human Use Guidelines for Good Clinical Practice. Informed consent was obtained from each patient after each was informed of the potential risks and benefits, as well as the investigational nature of each trial.

### Lurbinectedin plasma concentration measurement, ORR and OS definition, and neutrophil and platelet count determination

Total plasma concentrations of lurbinectedin were measured using a high-performance liquid chromatography tandem mass spectroscopy assay (in preparation). The lower limit of quantitation for the assay was set to 0.1 ng/mL. The within- and between-day precision ranged from 2.7 to 12.9% and from 5.1 to 10.7%, respectively. The within- and between-day accuracy (bias) ranged from − 10 to 12% and from − 5 to 6%, respectively. Additional details are provided in Supplementary Methods.

ORR was defined as the percentage of patients with complete or partial response defined per Investigator’s Assessment and confirmed by Independent Radiology review Committee, using RECIST v.1.1 [18]. Radiological tumor assessments were done at baseline, and every two cycles from the onset of the study treatment until cycle 6 or evidence of disease progression. After cycle 6, tumor assessment was performed every three cycles until evidence of disease progression. If an objective response was observed, according to the RECIST v.1.1, it had to be confirmed by the same method at least 4 weeks after the date of the first documentation of response. OS was defined as the time from the date of treatment to the date of death or last contact.

Total neutrophil and platelet counts were assessed using routine complete blood counts according to local site laboratories. NCI-CTCAE v.4. was used for the definition of G4 neutropenia and G ≥ 3 thrombocytopenia.

### Computer software

Datasets were prepared using SAS Enterprise Guide v.7.11 HF3 (SAS Institute Inc., Cary, NC, USA). Population PK assessment and exposure–response (ORR and myelosuppression) modeling were conducted using NONMEM v.7.3.0, SAEM with interaction, and IMP estimation methods (GloboMax LLC, Hanover, MD, USA). Compilations were achieved using gfortran v.4.8.5 (Free Software Foundation, Inc., Boston, MA, USA). Graphical and all other statistical analyses, including evaluation of NONMEM outputs, were performed with Perl speaks NONMEM v.4.6.0 [19], R v.3.2.5 (R Foundation for Statistical Computing, Vienna, Austria), and packages Xpose v.4.5.3 [20] and ggplot2 v.2.2.0 [21]. For OS, Cox proportional-hazards models and Kaplan–Meier plots were performed with SAS Enterprise Guide v.7.11 HF3.

### Pharmacokinetic analysis and exposure metrics

Lurbinectedin exposure during cycle 1 was selected for these exposure–response analyses because hematological toxicities were mostly observed in cycle 1 [16], and pharmacokinetics of lurbinectedin is not time-dependent [13]. Due to the low hepatic extraction ratio and high plasma protein binding of lurbinectedin, it was anticipated that unbound fraction of lurbinectedin was dependent on AAG and albumin levels and the AUC_*tot*_). Consequently, AUC_*tot*_ and AUC_*u*_ during cycle 1 were selected as exposure metrics.

AUC_*tot*_ was computed as dose/total clearance (CL_*tot*_), where empirical Bayesian estimates of individual CL_*tot*_ were obtained from the updated PopPK model. AUC_*u*_ was derived for individual patients from AUC_*tot*_, AAG and albumin concentrations, and respective dissociation rate constants (Eq. 1, [22]); where the dissociation constants for AAG (*K*_d1_ = 83.0 nM) and albumin (*K*_d2_ = 45,650 nM) were estimated in vitro by equilibrium dialysis,

1 $${AUC}_{u}=\frac{{AUC}_{tot}\times {K}_{d1}\times {K}_{d2}}{{L}_{1}\times AAG\times {K}_{d2}+{L}_{2}\times Albumin\times {K}_{d1}+{K}_{d1}\times {K}_{d2}},$$

where *L*_1_ and *L*_2_ are the scaling factors between lurbinectedin (MW 784.881) and both AAG (MW 68,000) and albumin (MW 42,000). *L*_1_ and *L*_2_ were fixed to 0.01869 (MW_lurbinectedin_/MW_AAG_) and 0.01154 (MW_lurbinectedin_/MW_Albumin_), respectively, assuming a 1:1 molar binding ratio for both proteins. Finally, AUC_*u*_ values were transformed from μg·h/L to ng·h/L to increase the magnitude of the odds ratio estimates.

### Exposure–response analyses

The primary efficacy endpoint of study B-005 was ORR by IRC. Radiological tumor assessments were done at baseline and every two cycles. CTFI (resistant disease [< 90 days] vs. sensitive disease [≥ 90 days]) was included as a prognostic factor, and OS was a secondary endpoint.

The E–R analysis of safety included G4 neutropenia as a primary endpoint since it is the main dose-limiting toxicity associated with lurbinectedin. G4 neutropenia events generally involve the administration of G-CSF and eventually evolve to febrile neutropenia, a life-threatening adverse event. G ≥ 3 thrombocytopenia was included as a secondary endpoint; although occurrences are less frequent than G4 neutropenia, thrombocytopenia may lead to bleeding events and involve the administration of platelet transfusions.

Multivariate analyses were performed to simultaneously incorporate into the models the relationship between AUC and the significant prognostic factor (i.e., CTFI), as well as describe the predictive factors (i.e., AAG, albumin and BSA) identified in previous exploratory and univariate analyses, which showed a better correlation of AUC_*u*_ than AUC_*tot*_. For E–R analysis of efficacy (ORR and OS), patients initially were classified into four equal-size groups based on the quartiles of AUC_*u*_. For ORR and safety outcomes, several mathematical models relating lurbinectedin AUC_*u*_ to the probability of response, G4 neutropenia and G ≥ 3 thrombocytopenia, were fitted in NONMEM. Initially, continuous AUC_*u*_ was assumed to be linearly related to the ORR logit and, if deemed appropriate, additional non-linear functions were used, including maximum lurbinectedin effect (*E*_max_) and sigmoid-*E*_max_ logistic regression models. Odds ratios for either event were calculated. The final E–R models of safety (G4 neutropenia and G ≥ 3 thrombocytopenia) were used to run simulations (*N* = 100 replicates) to predict the effect of dose regimen modifications. For OS, Cox proportional-hazards models relating AUC_*u*_ to the hazard of death were developed.

### Clinical utility index

The relationship between the probability of ORR and the probability of G4 neutropenia was estimated by means of a CUI [23], a quantitative method to determine the optimal trade-offs between key drug efficacy and safety profile, by bringing them into the same scale. Considering that therapeutic options are scarce for second-line SCLC with low probability of response and that G4 neutropenia is clinically manageable with secondary prophylaxis of G-CSF and dose reductions as needed, a 2:1 weighting scheme was used for ORR and incidence of G4 neutropenia, for a more realistic clinical benefit assessment.

## Results

### Pharmacokinetic analysis

The final model, using 9176 total plasma concentrations from 755 patients treated at a wide range of lurbinectedin doses (0.02–6.9 mg/m^2^) with two dose regimens (day 1 q3wk and days 1 and 8 q3wk), is described in Eqs. 2–6. The parameter estimates and bootstrap of the PopPK model, including the statistically significant covariate effects on model parameters, are presented in Online Resource Supplementary Table S2.

2 $${{\varvec{V}}}_{1,{\varvec{i}}}= {{\varvec{V}}}_{1} {\left(\frac{{AAG}_{i}}{121}\right)}^{{{\varvec{V}}}_{1,{\varvec{A}}{\varvec{A}}{\varvec{G}}}}\left(1+{{\varvec{V}}}_{1,{\varvec{A}}{\varvec{A}}{\varvec{G}}}\boldsymbol{ }\times \boldsymbol{ }\left({{\varvec{B}}{\varvec{S}}{\varvec{A}}}_{{\varvec{i}}}-1.76\right)\right){e}^{\eta {{\varvec{V}}}_{1}},$$

3 $${CL}_{i}= CL{\left(\frac{{AAG}_{i}}{121}\right)}^{{{\varvec{C}}{\varvec{L}}}_{{\varvec{A}}{\varvec{A}}{\varvec{G}}}}{\left(\frac{{ALB}_{i}}{4}\right)}^{{{\varvec{C}}{\varvec{L}}}_{{\varvec{A}}{\varvec{L}}{\varvec{B}}}}\left(1+{{\varvec{C}}{\varvec{L}}}_{{\varvec{I}}{\varvec{N}}{\varvec{H}}} \times INH\right){e}^{\eta CL},$$

4 $${V}_{3,i}= {{\varvec{V}}}_{3}{\left(\frac{{AAG}_{i}}{121}\right)}^{{{\varvec{V}}}_{3,{\varvec{A}}{\varvec{A}}{\varvec{G}}}}{\left(\frac{{BSA}_{i}}{1.76}\right)}^{{{\varvec{V}}}_{3,{\varvec{A}}{\varvec{A}}{\varvec{G}}}}\left(1+{{\varvec{V}}}_{3,{\varvec{S}}{\varvec{E}}{\varvec{X}}{\varvec{F}}} \times SEXF\right){e}^{\eta {{\varvec{V}}}_{3}},$$

5 $${Q}_{3,i}= {{\varvec{Q}}}_{3}{\left(\frac{{AAG}_{i}}{121}\right)}^{{{\varvec{Q}}}_{3,{\varvec{A}}{\varvec{A}}{\varvec{G}}}}{\left(\frac{{BSA}_{i}}{1.76}\right)}^{{{\varvec{Q}}}_{3,{\varvec{B}}{\varvec{S}}{\varvec{A}}}}\left(1+{{\varvec{Q}}}_{3,{\varvec{S}}{\varvec{E}}{\varvec{X}}{\varvec{F}}} \times SEXF\right){e}^{\eta {{\varvec{Q}}}_{3}},$$

6 $${V}_{2,i}= {{\varvec{V}}}_{2}{\left(\frac{{{\varvec{A}}{\varvec{A}}{\varvec{G}}}_{i}}{121}\right)}^{{{\varvec{V}}}_{2,{\varvec{A}}{\varvec{A}}{\varvec{G}}}}\left(1+{{\varvec{V}}}_{2,{\varvec{B}}{\varvec{S}}{\varvec{A}}}\boldsymbol{ }\times \boldsymbol{ }\left({{\varvec{B}}{\varvec{S}}{\varvec{A}}}_{{\varvec{i}}}-1.76\right)\right){e}^{\eta {{\varvec{V}}}_{2}}.$$

An open three-compartment model with linear elimination was able to describe the time course of total plasma concentration and its variability. An initial rapid distribution half-life (*t*_*1/2*_) of about 8–9 min is followed by a slower *t*_*1/2*_ of about 1.9 h and a terminal *t*_*1/2*_ of about 51 h, with the latter phase constituting a substantial portion of the overall AUC. In study B-005, the median (range) of estimated lurbinectedin AUC_*tot*_ and AUC_*u*_ at 3.2 mg/m^2^ in study B-005 were 493 (176–1083) μg·h/L and 1400 (433–3136) ng·h/L, respectively.

### Exposure–response analysis

#### Objective response rate

**Fig. 1:**
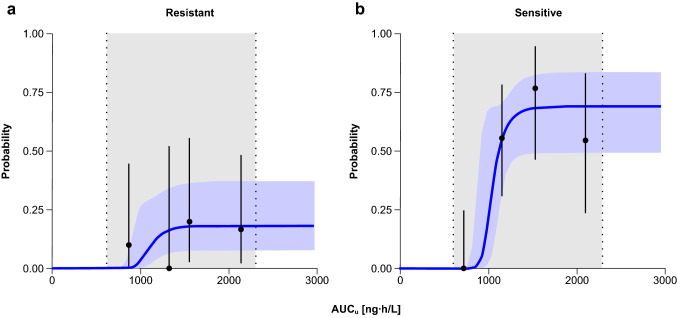
Relationship between ORR by IRC and AUCu stratified by CTFI. Solid black dots represent the proportion of responders grouped by quartiles of AUC_*u*_ and plotted at the median AUC_*u*_ for each quartile in resistant (**a**) and sensitive (**b**) patients. Bars represent the 95% CI for the proportion of each quartile. Curve and blue shaded area represent predicted values and 95% CI of model-predicted ORR, respectively. The vertical point lines and the gray shaded area represent the 95% prediction interval of the observed AUC_*u*_. *AUC*_*u*_ unbound plasma area under the concentration–time curve, *CI* confidence interval, *CTFI* chemotherapy-free interval, *IRC* independent review committee, *ORR* objective response rate

Among several linear and non-linear models examined, a sigmoid-*E*_max_ model for AUC_*u*_, where CTFI modified the *E*_max_ parameter (Eq. 7), was found to best describe ORR data.

7 $${\varvec{l}}{\varvec{o}}{\varvec{g}}{\varvec{i}}{\varvec{t}}\boldsymbol{ }\left({\varvec{O}}{\varvec{R}}{\varvec{R}}\right)=\boldsymbol{ }-10+\frac{{{\varvec{E}}}_{{\varvec{m}}{\varvec{a}}{\varvec{x}}}\boldsymbol{ }\times {{\varvec{A}}{\varvec{U}}{\varvec{C}}}_{{\varvec{u}}}^{10}\boldsymbol{ }}{\boldsymbol{ }{{\varvec{E}}{\varvec{C}}}_{50}^{10}\boldsymbol{ }+\boldsymbol{ }{{\varvec{A}}{\varvec{U}}{\varvec{C}}}_{{\varvec{u}}}^{10}},$$

where EC_50_ was the AUC_*u*_ that provided half *E*_max_ on ORR. Parameter estimates with relative standard errors (RSEs) in the final model are shown in Table 1. Observed and model-predicted ORRs by IRC, stratified by CTFI, are depicted in Fig. 1. Figure 1 Relationship between ORR by IRC and AUCu stratified by CTFI; the maximum ORRs for patients with sensitive and resistant SCLC were 69.1% (95% CI 49.3–83.8) and 18.1% (95% CI 7.7–37.1), respectively, reaching its 95% CI with AUC_*u*_ above 1337 and 1433 ng·h/L.

**Table 1:** Parameter estimates and their uncertainty for the final ORR, G4 neutropenia and G ≥ 3 thrombocytopenia logistic regression models

Parameter	Estimate (RSE%)
ORR model	
*E*_max_ resistant	8.49 (5.88)
*E*_max_ sensitive	10.81 (3.94)
EC_50_ (ng·h/L)	877 (7.73)
G4 neutropenia model based on AUC_*u*_	
*E*_max_	9.28 (3.23)
EC_50_ (ng·h/L)	65.4 (35.0)
G4 neutropenia model based on AUC_*tot*_	
*E*_max_	11.4 (4.18)
EC_50_ (μg·h/L)	561 (17.6)
G ≥ 3 thrombocytopenia model based on AUC_*u*_	
Baseline	− 4.61 (8.32)
Slope_AUCu_ (ng·h/L)	0.000873 (19.9)
Slope_Platelets_	− 0.01 (41.9)
Slope_BSA_	− 2.58 (31.9)
Slope_Albumin_	− 1.04 (36.9)
G ≥ 3 thrombocytopenia model based on AUC_*tot*_	
Baseline	− 3.96 (10.1)
Slope_AUCu_ (μg·h/L)	0.00136 (34.2)
Slope_Platelets_	− 0.00635 (45.5)
Slope_BSA_	− 2.31 (32.9)
Slope_Albumin_	− 1.23 (35.8)
Slope_AAG_	− 0.00911 (45.5)

*AAG* α-1-acid glycoprotein, *AUC*_*tot*_ total plasma area under the concentration–time curve, *AUC*_*u*_ unbound plasma area under the concentration–time curve, *BSA* body surface area, *E*_*max*_ maximal response, *EC*_*50*_ half maximal effective concentration, *G* grade, *ORR* objective response rate, *SE* relative standard error

#### Overall survival

**Fig. 2:**
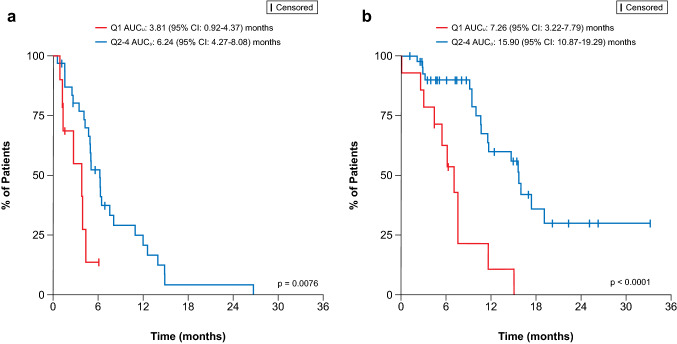
Kaplan–Meier plots for OS versus pooled AUC_*u*_ (first vs. second to fourth quartiles), stratified by CTFI (**a**, resistant; **b**, sensitive). Q1 AUC_*u*_: first quartile of AUC_*u*_; Q2–4 AUC_*u*_: second to fourth quartiles of AUC_*u*_. *AUC*_*u*_ unbound plasma area under the concentration–time curve, *CTFI* chemotherapy-free interval, *OS* overall survival

Based on the differences between first quartile and upper quartiles of AUC_*u*_ and differences in OS between sensitive and resistant disease, upper quartiles (second to fourth) were pooled and compared against the lowest quartile and stratified by CTFI (Fig. 2). In patients with resistant and sensitive disease, pooled AUC_*u*_ (first vs. second to fourth quartiles) showed statistically significant differences. Median overall survival in patients with resistant disease were 3.81 months (95% CI 0.92–4.37) and 6.24 months (95% CI 4.27–8.08) for first and upper quartiles, respectively, after the start of lurbinectedin treatment, while in patients with sensitive disease were 7.26 months (95% CI 3.22–7.79) and 15.90 months (95% CI 10.87–19.29), respectively (Fig. 2). Additionally, in a multivariate Cox regression analysis including CTFI and AUC_*u*_ as a continuous variable, relationships were found for both CTFI and AUC_*u*_, with hazard ratios of 0.22 (95% CI 0.12–0.39) and 0.41 (95% CI 0.23–0.72), respectively (Table 2). This translates to a 4.6-fold higher risk of death, and a 2.5-fold lower risk of death per unit increase in AUC_*u*_ (μg·h/L), in patients with resistant disease than in patients with sensitive disease.

**Table 2:** Cox regression analysis with OS by CTFI and AUC_*u*_

Parameter		DF	Parameter estimate	Standard error	Chi-square	Pr > ChiSq	Hazard ratio	95% CI
CTFI	2	1	− 1.52	0.29	26.74	< 0.0001	0.22	(0.12–0.39)
AUC_*u*_		1	− 0.90	0.29	9.52	0.002	0.41	(0.23–0.72)

*AUC*_*u*_, unbound plasma area under the concentration–time curve, *ChiSq* Chi-square, *CTFI* chemotherapy-free interval, *DF* degrees of freedom, *OS* overall survival, *Pr > ChiSq* associated *p* value

#### G4 neutropenia

Several covariates were explored to assess their impact on G4 neutropenia: dose, neutrophils at baseline, albumin, AAG, and BSA. After a number of models were tested using continuous metrics for exposure, an *E*_max_ model based on changes of AUC_*u*_, where the base parameter was fixed to a low value (− 10), was finally selected. An equivalent model with AUC_*tot*_ presented higher objective function value (ofv), although this improved when AAG was included.

8 $${\varvec{l}}{\varvec{o}}{\varvec{g}}{\varvec{i}}{\varvec{t}}\left({\varvec{n}}{\varvec{e}}{\varvec{u}}{\varvec{t}}{\varvec{r}}{\varvec{o}}{\varvec{p}}{\varvec{e}}{\varvec{n}}{\varvec{i}}{\varvec{a}}\boldsymbol{ }{\varvec{g}}{\varvec{r}}{\varvec{a}}{\varvec{d}}{\varvec{e}}\boldsymbol{ }4\right)={\varvec{B}}{\varvec{a}}{\varvec{s}}{\varvec{e}}+\frac{{{\varvec{E}}}_{{\varvec{m}}{\varvec{a}}{\varvec{x}}}\times {\varvec{A}}{\varvec{U}}{\varvec{C}}}{{{\varvec{E}}{\varvec{C}}}_{50}+{\varvec{A}}{\varvec{U}}{\varvec{C}}}$$

**Fig. 3:**
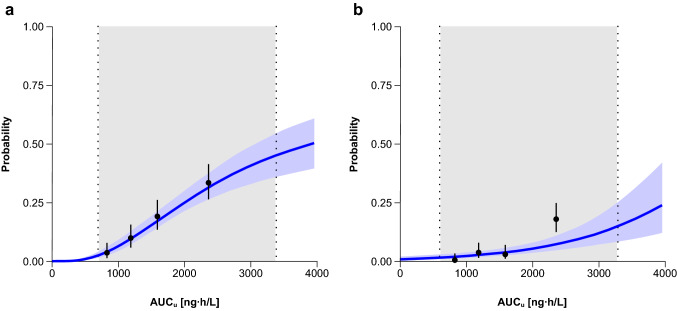
Relationship between AUC_*u*_ and G4 neutropenia (**a**) and G ≥ 3 thrombocytopenia (**b**). Solid black dots represent the G4 neutropenia or G ≥ 3 thrombocytopenia incidence grouped by quartiles of AUC_*u*_ and plotted at the median AUC_*u*_ for each quartile. The bars represent the 95% confidence interval for the proportion of each quartile. Curve and blue shaded area represent predicted values and 95% confidence intervals of model-predicted risk of G4 neutropenia or G ≥ 3 thrombocytopenia, respectively. The vertical point lines and the gray shaded area represent the 95% prediction interval of the observed AUC_*u*_ in patients treated at doses from 0.02 to 6.9 mg/m^2^. *AUC*_*u*_ unbound plasma area under the concentration–time curve, *G* grade

Figure 3 shows the probability of G4 neutropenia predicted by the model for AUC_*u*_. Parameter estimates with RSE in the final models based on AUC_*u*_ and AUC_*tot*_ are shown in Table 1.

AUC_*u*_ from study B-005 was bootstrapped to predict the incidence of G4 neutropenia at doses of 3.2, 2.6, and 2.0 mg/m^2^, which correspond to the recommended dose, the first and second 20% dose reductions, as recommended in the label. The resulting decreases in lurbinectedin exposure following the first and second 20% dose reductions are expected to lower the incidence of G4 neutropenia from the 16.5% (95% CI 16.4–16.6) incidence at 3.2 mg/m^2^ to 11.3% (95% CI 11.2–11.4), and 7.5% (7.4–7.7), at 2.6, and 2.0 mg/m^2^.

#### G ≥ 3 thrombocytopenia

Several models for G ≥ 3 thrombocytopenia exploring a number of covariates (i.e., dose, platelets at baseline, albumin, AAG, BSA) were examined. A linear logistic model based on AUC_*u*_ showed the best fit, where higher values of retained covariates reduced the likelihood of G ≥ 3 thrombocytopenia.

9 $${\varvec{l}}{\varvec{o}}{\varvec{g}}{\varvec{i}}{\varvec{t}}\left({\varvec{t}}{\varvec{h}}{\varvec{r}}{\varvec{o}}{\varvec{m}}{\varvec{b}}{\varvec{o}}{\varvec{c}}{\varvec{y}}{\varvec{t}}{\varvec{o}}{\varvec{p}}{\varvec{e}}{\varvec{n}}{\varvec{i}}{\varvec{a}}\boldsymbol{ }{\varvec{g}}{\varvec{r}}{\varvec{a}}{\varvec{d}}{\varvec{e}}\ge 3\right)={\varvec{B}}{\varvec{a}}{\varvec{s}}{\varvec{e}}{\varvec{l}}{\varvec{i}}{\varvec{n}}{\varvec{e}}+{{\varvec{S}}{\varvec{l}}{\varvec{o}}{\varvec{p}}{\varvec{e}}}_{{{\varvec{A}}{\varvec{U}}{\varvec{C}}}_{{\varvec{u}}}}\times {{\varvec{A}}{\varvec{U}}{\varvec{C}}}_{{{\varvec{u}}}_{{\varvec{i}}}}+{{\varvec{S}}{\varvec{l}}{\varvec{o}}{\varvec{p}}{\varvec{e}}\boldsymbol{ }}_{{\varvec{P}}{\varvec{l}}{\varvec{a}}{\varvec{t}}{\varvec{e}}{\varvec{l}}{\varvec{e}}{\varvec{t}}{\varvec{s}}}\times \boldsymbol{ }\left({{\varvec{P}}{\varvec{l}}{\varvec{a}}{\varvec{t}}{\varvec{e}}{\varvec{l}}{\varvec{e}}{\varvec{t}}{\varvec{s}}}_{{\varvec{i}}}\boldsymbol{ }\boldsymbol{ }-243\right)+{{\varvec{S}}{\varvec{l}}{\varvec{o}}{\varvec{p}}{\varvec{e}}}_{{\varvec{B}}{\varvec{S}}{\varvec{A}}}\times \boldsymbol{ }\left({{\varvec{B}}{\varvec{S}}{\varvec{A}}}_{{\varvec{i}}}-1.75\right)+\boldsymbol{ }{{\varvec{S}}{\varvec{l}}{\varvec{o}}{\varvec{p}}{\varvec{e}}}_{{\varvec{A}}{\varvec{l}}{\varvec{b}}{\varvec{u}}{\varvec{m}}{\varvec{i}}{\varvec{n}}}\times \left({{\varvec{A}}{\varvec{l}}{\varvec{b}}{\varvec{u}}{\varvec{m}}{\varvec{i}}{\varvec{n}}}_{{\varvec{i}}}-4.0\right),$$

where ${AUC}_{{u}_{i}}$, *Platelets*_*i*_, *BSA*_*i*_, and *Albumin*_*i*_ correspond to the individual values for AUC_*u*_, platelets, BSA, and albumin at baseline, respectively, for the *i*th individual. An equivalent model based on AUC_*tot*_ showed a higher ofv, which improved when AAG was added (see Table 1).

Since BSA was not associated to *CL* in the PopPK model, although it did to *V*_1_, *V*_2_, *V*_3_, and *Q*_3_, confounding factors causing the effect of BSA on thrombocytopenia were explored. In this sense, factors included in a previously developed semi-mechanistic PKPD model of lurbinectedin on platelets [16] were analyzed. Apart from BSA (affecting EC_50_), baseline platelets (as part of the structural model) and tumor type (affecting EC_50_), with pancreas and ovarian as more sensitive tumors, were the detected covariates. BSA was neither related with baseline platelets nor with tumor type, so confounders affecting the relationship between BSA and thrombocytopenia are unlikely.

As described above for neutropenia, AUC_*u*_ from study B-005 was bootstrapped to predict the incidence of G ≥ 3 thrombocytopenia at doses of 3.2, 2.6, and 2.0 mg/m^2^. The resulting decreases in lurbinectedin exposure following the first and second 20% dose reductions are expected to lower the incidence of G4 neutropenia from the 4.8% (95% CI 4.7–4.9) incidence at 3.2 mg/m^2^ to 3.5% (95% CI 3.4–3.5), and 2.75% (2.6–2.7), at 2.6, and 2.0 mg/m^2^.

As BSA was retained in the model, the incidence of G ≥ 3 thrombocytopenia at a fixed dose of 5.6 mg (equivalent to 3.2 mg/m^2^ for a BSA of 1.75 m^2^) increased a 16% in patients lower BSA. Less variation in the incidence of G ≥ 3 thrombocytopenia was observed with BSA-based dosing: 6.4% (95% CI 5.3–8.1) in patients with lower BSA (< 1.64 m^2^) and 3.1% (95% CI 1.9–4.8) in patients with larger BSA; with FD, the incidences were 7.6% (95% CI 6.2–9.5) and 2.5% (95% CI 1.6–4.0), respectively.

#### Clinical utility index

**Fig. 4:**
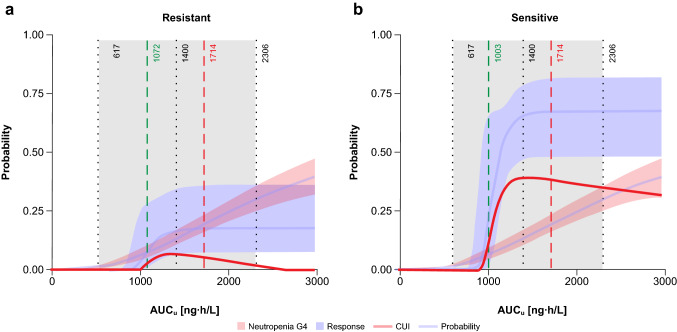
Clinical utility index with AUC_*u*_ in resistant (**a**) and sensitive (**b**) SCLC patients**.** Dashed green vertical line is the lurbinectedin AUC_*u*_ providing an ORR of 7.5% (resistant) and 19.3% (sensitive), which are the ORRs corresponding to topotecan. Dashed dark red vertical line is the AUC_*u*_ at which the probability of grade 4 neutropenia is 20%. The gray shaded area represents the 95% prediction interval of the observed AUC_*u*_ in SCLC patients treated at 3.2 mg/m^2^. Black dotted vertical lines are percentiles 5, 50, and 95 of AUC_*u*_. *AUC*_*u*_ unbound plasma area under the concentration–time curve, *CUI* clinical utility index, *G* grade, *SCLC* small-cell lung cancer

To depict the adequacy of the dose regimen of 3.2 mg/m^2^ q3wk in terms of the relationship between the probability of ORR and the probability of G4 neutropenia, a CUI was estimated (Fig. 4). The best benefit/risk ratio appeared at lurbinectedin AUC_*u*_ between approximately 1000 and 1700 ng·h/L.

## Discussion

This integrated E-R analysis indicates that lurbinectedin exposure was significantly associated with efficacy and safety outcomes. Maximal ORR and OS were found in patients with AUC_*u*_ from approximately 1400 ng·h/L and 1000 ng·h/L, respectively, which may reveal that ORR is a more treatment directly linked outcome and, therefore, more AUC_*u*_ dependent, while OS may be more affected by other factors after treatment with lurbinectedin. The magnitude of lurbinectedin exposure effect on ORR (see Fig. 1) and OS see (Fig. 2) was dependent on CTFI. For patients with sensitive disease, the maximal ORR and the median survival were estimated to be 69.1% and 15.9 months for lurbinectedin AUC_*u*_ above 1337 ng·h/L and 1052 ng·h/L, respectively. However, for patients with resistant disease, the ORR and the median OS were estimated to be 18.1% and 6.2 months for AUC_*u*_ above 1433 ng·h/L and 1007 ng·h/L, respectively.

Historical efficacy data from seven published clinical trials with topotecan in second-line SCLC (Online Resource Supplementary Table S3) suggest mean ORRs of 7.5% (95% CI 4.8–10.9, *n* = 322) and 19.3% (95% CI 15.1–24.0, *n* = 327) for resistant and sensitive SCLC patients, respectively. Therefore, based on these point estimates, lurbinectedin AUC_*u*_ above 1072 ng·h/L and 1003 ng·h/L, which are similar to EC_50_ of ORR (Table 1), would improve the topotecan ORR, up to 18.1% and 69.1%, for resistant and sensitive patients, respectively (see Fig. 4).

In terms of safety outcomes, the present analysis provides a robust relationship between lurbinectedin exposure and the probability of myelosuppression (see Fig. 3). Lurbinectedin AUC_*u*_ above 1714 ng·h/L increased the incidence of G4 neutropenia beyond 20%. One or two sequential 20% dose reductions during the course of treatment with lurbinectedin are recommended in the label to manage severe episodes of neutropenia or thrombocytopenia and improve lurbinectedin tolerability in patients who develop these toxicities after lurbinectedin treatment. The first dose reduction resulted in a G4 neutropenia and G ≥ 3 thrombocytopenia incidence relative decrease of 32% and 27%, respectively. A second 20% dose reduction produced an additional decrease of 34% and 21%.

Moreover, the BSA-based dosing at 3.2 mg/m^2^ provides a 16% reduction in the incidence of severe thrombocytopenia in patients with BSA < 1.65 m^2^, thus favoring the use of BSA-based dosing over flat dosing.

Consequently, AUC_*u*_ between approximately 1000 and 1700 ng·h/L provided the best benefit/risk ratio for lurbinectedin, and the recommended dosing regimen of 3.2 mg/m^2^ provided a median AUC_*u*_ of 1400 ng·h/L, which maximizes the proportion of patients within this lurbinectedin target exposure range. Lowering the dose resulted in a dramatic drop in efficacy, whereas increasing the dose increased the incidence of severe hematological toxicity without apparent improvement in efficacy (see Fig. 4).

The main limitation of this study is that the exposure–efficacy analysis relies on a unique dose level. Nevertheless, a clear relationship between AUC_*u*_ and ORR was characterized, which was further endorsed by the relationship between the same exposure metric and OS. Another potential limitation is the lack of direct in vivo quantification of unbound lurbinectedin plasma concentrations, and, therefore, the estimation of individual AUC_*u*_ was based on the AUC_*tot*_, AAG, and albumin from each patient. In a published PopPK model, a reduction in total plasma CL associated with increased AAG and CRP and reduced albumin was detected [13]. Additional experiments were planned to elucidate the underlying reason of such an inflammatory pattern leading to a decreased CL. An in vitro plasma protein-binding study shed light in revealing the preferential affinity of lurbinectedin to AAG, and dissociation constants for AAG and albumin could be calculated to allow AUC_*u*_ estimation. Unbound lurbinectedin plasma concentrations are being measured with a validated method in ongoing drug-drug interaction and organ impairment studies, so that a comparison between model-based estimated AUC_*u*_ and observed AUC_*u*_ can be made.

In this sense, the safety models presented herein established E–R relationships with both AUC_*u*_ and AUC_*tot*_, by virtue of a large database of patients (*n* = 755) treated at a wide range of lurbinectedin doses, although model diagnostics showed a better fit of AUC_*u*_ over AUC_*tot*_. However, the efficacy model could not be fitted to AUC_*tot*_, but only to AUC_*u*_. Given the low extraction ratio, lurbinectedin AUC_*tot*_ and unbound fraction were interfered by plasma protein levels, which vary substantially among cancer patients, while AUC_*u*_ remained unaffected [24]. AUC_*u*_ is more representative of active lurbinectedin AUC, thus exposing existing relationships with efficacy outcomes, regardless of the smaller efficacy database of patients (*n* = 99) treated at a single dose level.

## Conclusion

The relationships evidenced in this integrated E–R analysis, with efficacy variables at a single dose level and safety variables at a wide range of doses, support a favorable benefit-risk profile for the approved lurbinectedin dose regimen of 3.2 mg/m^2^ q3wk. One or two sequential 20% dose reductions during the course of lurbinectedin treatment are adequate to manage patients who develop severe episodes of myelosuppression.

## Supplementary Information

Below is the link to the electronic supplementary material.

Supplementary file1 (PDF 202 KB)

Supplementary file2 (DOCX 13 KB)

## Data Availability

Qualified researchers may request access to data by contacting the corresponding author.

## Code availability

Qualified researchers may request access to model codes by contacting the corresponding author.
