# Supplementary material for: Integrated exposure–response analysis of efficacy and safety of lurbinectedin to support the dose regimen in small-cell lung cancer
Source: Cancer Chemother Pharmacol. 2021 Nov 5;89(5):585–94. doi: 10.1007/s00280-021-04366-3 (PMC9054899; doi:10.1007/s00280-021-04366-3)
Supplement: Supplementary file 1 — Supplementary file1 (PDF 202 KB) [file 280_2021_4366_MOESM1_ESM.pdf]

Integrated Exposure-Response Analysis of Efficacy and Safety of Lurbinectedin to Support the Dose Regimen in Small Cell Lung Cancer

Carlos Fernández-Teruel, Salvador Fudio, Rubin Lubomirov

Pharma Mar, Colmenar Viejo, Madrid, Spain

Short title: Lurbinectedin exposure-response in SCLC

ORCID codes:

Carlos Fernández-Teruel: 0000-0002-1473-2267

Salvador Fudio: 0000-0001-7320-6139

Rubin Lubomirov: 0000-0003-0550-8228

Corresponding author:

Rubin Lubomirov, M.D., Ph.D.

Pharma Mar, S.A.

Avda. De los Reyes, 1, Pol. Ind. La Mina-Norte

28770 Colmenar Viejo, Madrid, Spain

Phone: +34-918234565

Fax: +34-918466001

E-mail: rlubomirov@pharmamar.com

Supplementary Information

Supplementary Table S1 Characteristics of the clinical trials included in the integrated E-R analysis of lurbinectedin

Study	Dose level	Tumor types	Patients treated	Patients with ORR data	Patients with PK and safety data	ANC/platelets sampling schedule
PM1183-A-001-08 NCT00877474 [1]	0.02–6.9 mg/m² (D1)	Advanced solid tumors	33	NA	31	D1, D8, D15, and D22 of every cycle, and on screening, end of treatment, and follow-up
PM1183-A-002-10[a] NCT01314599 [2]	3.5–7.0 mg FD (D1,8)	Hematological malignancies	24	NA	23	NA
	1.0–3.0 mg FD (D1-3)		18	NA	18	
PM1183-A-005-11[a] NCT01405391	3.0–5.0 mg FD (D1,8)	Advanced solid tumors	21	NA	21	NA
PM1183-B-001-10	7.0 mg FD (D1)	Pancreatic cancer	45	NA	44	D8 and D15 of cycle 1, D1 and D10 of further cycles, and on screening and end of treatment
PM1183-B-002-10 [4]	7.0 mg FD (D1)	Ovarian cancer	52	NA	22	D8 and D15 of cycle 1, D1 and D10 of further cycles, and on screening and end of treatment
PM1183-B-003-11 NCT01525589 [5]	7.0 mg FD (D1)	Breast cancer	109	NA	38	D8 and D15 of cycle 1, D1 and D10 of cycle 2, D1 of further cycles, and on screening and end of treatment. From cycle 2 onwards, D10 only if toxicity
PM1183-B-004-13 NCT01951157	7.0 mg FD & 3.2 mg/m² (D1)	NSCLC	21	NA	21	D8 and D15 of cycle 1, D1 and D8 of further cycles, and on screening and end of treatment
PM1183-B-005-14 NCT02454972 [6]	3.2 mg/m² (D1)	Selected solid tumors	335[b]	96	333	D1, D8, and 15 during cycles 1 and 2, D1 of further cycles, and screening and end of treatment. From cycle 2 onwards, D8 and D15 only if toxicity

PM1183-C-004-14 NCT02421588 [7]	3.2 mg/m² (D1)	Ovarian cancer	219	NA	204	Screening and D8 of cycle 1, D1 and D8 of cycle 2, and D1 of further cycles.

ANC, absolute neutrophil count; D, day; E-R, exposure-response; FD, flat dose; NA, not applicable; NSCLC, non–small cell lung cancer; ORR, objective response rate; PK, pharmacokinetics.

[a]Included in pool of population PK analysis. [b]SCLC cohort in B-005 study included 105 patients.

Supplementary Table S1 References

1. Elez ME, Tabernero J, Geary D, Macarulla T, Kang SP, Kahatt C, Pita AS, Teruel CF, Siguero M, Cullell-Young M, Szyldergemajn S, Ratain MJ (2014) First-in-human phase I study of Lurbinectedin (PM01183) in patients with advanced solid tumors. Clin Cancer Res 20 (8):2205-2214. doi:http://doi.org/10.1158/1078-0432.CCR-13-1880
2. Benton CB, Rodriguez-Diaz-Pavon J, Maiti A, Daver NG, Ravandi F, Jain N, Alvarado Y, Jabbour E, Pierce S, Kwari M, Santos MA, Martinez S, Siguero M, Tefferi A, Cortes JE, Kantarjian HM, Pardanani AD, Garcia-Manero G (2017) Phase I study of lurbinectedin (PM11083) in patients with advanced AML and MDS. Journal of Clinical Oncology 35 (15_suppl):e18521-e18521. doi:http://doi.org/10.1200/JCO.2017.35.15_suppl.e18521
3. Jimeno A, Sharma MR, Szyldergemajn S, Gore L, Geary D, Diamond JR, Fernandez Teruel C, Soto Matos-Pita A, Iglesias JL, Cullell-Young M, Ratain MJ (2017) Phase I study of lurbinectedin, a synthetic tetrahydroisoquinoline that inhibits activated transcription, induces DNA single- and double-strand breaks, on a weekly x 2 every-3-week schedule. Investigational new drugs 35 (4):471-477. doi:http://doi.org/10.1007/s10637-017-0427-2
4. Poveda A, Del Campo JM, Ray-Coquard I, Alexandre J, Provansal M, Guerra Alia EM, Casado A, Gonzalez-Martin A, Fernandez C, Rodriguez I, Soto A, Kahatt C, Fernandez Teruel C, Galmarini CM, Perez de la Haza A, Bohan P, Berton-Rigaud D (2017) Phase II randomized study of PM01183 versus topotecan in patients with platinum-resistant/refractory advanced ovarian cancer. Annals of oncology : official journal of the European Society for Medical Oncology / ESMO 28 (6):1280-1287. doi:http://doi.org/10.1093/annonc/mdx111
5. Cruz C, Llop-Guevara A, Garber JE, Arun BK, Perez Fidalgo JA, Lluch A, Telli ML, Fernandez C, Kahatt C, Galmarini CM, Soto-Matos A, Alfaro V, Perez de la Haza A, Domchek SM, Antolin S, Vahdat L, Tung NM, Lopez R, Arribas J, Vivancos A, Baselga J, Serra V, Balmana J, Isakoff SJ (2018) Multicenter phase II study of lurbinectedin in BRCA-mutated and unselected metastatic advanced breast cancer and biomarker assessment substudy. J Clin Oncol 36 (31):3134-3143. doi:http://doi.org/10.1200/JCO.2018.78.6558
6. Trigo J, Subbiah V, Besse B, Moreno V, Lopez R, Sala MA, Peters S, Ponce S, Fernandez C, Alfaro V, Gomez J, Kahatt C, Zeaiter A, Zaman K, Boni V, Arrondeau J, Martinez M, Delord JP, Awada A, Kristeleit R, Olmedo ME, Wannesson L, Valdivia J, Rubio MJ, Anton A, Sarantopoulos J, Chawla SP, Mosquera-Martinez J, D'Arcangelo M, Santoro A, Villalobos VM, Sands J, Paz-Ares L (2020)

Lurbinectedin as second-line treatment for patients with small-cell lung cancer: a single-arm, open-label, phase 2 basket trial. The Lancet Oncology 21 (5):645-654. doi:http://doi.org/10.1016/S1470-2045(20)30068-1
7. Leary A, Gaillard S, Vergote I, Trigo J, Kahatt C, Nieto A, Fernandez CM, Cullell-Young M, Zeaiter AH, Subbiah V Pooled safety analysis of single-agent lurbinectedin versus topotecan (Results from a randomized phase III trial CORAIL and a phase II basket trial). ASCO American Society of Clinical Oncology, 56th Annual Meeting, virtual, 31 May-Jun 4, 2020 J Clin Oncol

Supplementary Table S2 Summary of pharmacokinetic parameters in final model and bootstrap results

Parameter	Final model estimate (RSE%)	Non-parametric bootstrap (n=400)	
		Median (RSE%)	95% CI
Typical parameter			
V_1 (L)	12.8 (3.80)	12.4 (3.99)	11.3–13.4
CL (L/h)	10.6 (2.25)	10.5 (2.21)	9.95–10.9
V_3 (L)	454 (2.26)	447 (3.16)	424–480
Q_3 (L/h)	16.0 (2.05)	15.9 (2.36)	15.2–16.7
V_2 (L)	37.5 (2.50)	37.0 (2.24)	35.5–38.7
Q_2 (L/h)	31.8 (2.38)	31.7 (3.13)	29.9–33.9
Residual variability (CV%)			
RV	31.7 (2.52)	30.9 (2.39)	29.4–32.5
Inter-individual variability (CV%)			
η V_1	34.6 (13.2)	31.7 (31.2)	21.7–41.3
η CL	49.9 (3.96)	50.0 (9.42)	45.7–54.4
η V_3	39.1 (5.33)	37.2 (31.2)	31.5–45.5
η Q_3	27.2 (6.54)	27.0 (19.1)	24.2–30.1
η V_2	33.4 (7.78)	30.7 (15.7)	25.6–35.0
η RV	59.2 (3.42)	59.6 (15.7)	55.0–63.6
η AAG_{C004}	54.2 (13.6)	47.1 (27.7)	35.7–60.3
Inter-individual variability correlation (%)			
η Q_3 – η V_3	77.0 (6.42)	75.7 (13.2)	77.5–72.4
Typical covariate parameters			
CL_{AAG}	-0.627 (8.36)	-0.727 (10.3)	-0.858 to -0.568
CL_{ALB}	0.746 (19.6)	0.633 (28.2)	0.261–0.979
CL_{INH}	-0.408 (12.2)	-0.407 (14.6)	-0.517 to -0.275
$Q_{3,AAG}$	-0.578 (8.57)	-0.603 (8.18)	-0.697 to -0.504
$Q_{3,BSA}$	0.990 (14.2)	0.964 (15.3)	0.658–1.227
$Q_{3,SEXF}$	-0.195 (14.4)	-0.198 (16.3)	-0.254 to -0.13
$V_{1,AAG}$	-0.992 (9.78)	-1.032 (9.83)	-1.233 to -0.839
$V_{2,AAG}$	-0.653 (10.6)	-0.675 (9.99)	-0.803 to -0.541
$V_{2,BSA}$	0.423 (25.6)	0.390 (28.5)	0.188– 0.630
$V_{3,AAG}$	-0.517 (11.8)	-0.600 (17.7)	-0.758 to -0.319
$V_{3,BSA}$	1.915 (8.70)	1.802 (10.1)	1.474–2.174
$V_{3,SEXF}$	-0.244 (13.1)	-0.245 (15.1)	-0.312 to -0.174
$V_{1,BSA}$	0.748 (21.3)	0.744 (23.1)	0.436–1.129
AAG_{C004}	260 (7.53)	230 (9.42)	201–280

AAG, α-1-acid glycoprotein; AAG_{C004}, AAG in study C-004 CORAIL; ALB, albumin; BSA, body surface area; 95% CI, 95% confidence interval; CL, clearance; CL_{AAG}, relationship between CL and AAG; CL_{ALB}, relationship between CL and albumin; CL_{INH}, relationship between CL and CYP3A inhibitors; CV, coefficient of variation; INH, CYP3A inhibitor; Q_2, intercompartmental clearance for shallow compartment; Q_3, intercompartmental clearance for deep compartment; $Q_{3,AAG}$, relationship between Q_3 and AAG; $Q_{3,BSA}$, relationship between Q_3 and BSA; $Q_{3,SEXF}$, relationship between Q_3 and gender; RSE, relative standard error; RV, residual variability; SEXF, gender; V_1,

apparent volume of distribution of central compartment; V_2, apparent volume of distribution of shallow peripheral compartment; V_3, apparent volume of distribution of deep peripheral compartment; $V_{1,AAG}$, relationship between V_1 and AAG; $V_{1,BSA}$, relationship between V_1 and BSA; $V_{2,AAG}$, relationship between V_2 and AAG; $V_{2,BSA}$, relationship between V_2 and BSA; $V_{3,AAG}$, relationship between V_3 and AAG; $V_{3,BSA}$, relationship between V_3 and BSA; $V_{3,SEXF}$, relationship between V_3 and gender.

Supplementary Table S3 ORR of topotecan in clinical trials in patients with second-line SCLC

Study	Resistant (CTFI < 90 days)			Sensitive (CTFI ≥ 90 days)		
	Responders (n)	Total patients (n)	ORR (%) (95% CI)	Responders (n)	Total patients (n)	ORR (%) (95% CI)
von Pawel (1999) [1]	3	22	13.64	0	0	0
O'Brien (2006) [2]	4	41	9.76	1	30	3.33
von Pawel (2014) [3]	9	96	9.38	25	117	21.37
Evans (2015) [4]	3	37	8.11	5	42	11.9
Ardizzoni (1997) [5]	3	47	6.38	17	45	37.78
Depierre (1997) [6]	1	41	2.44	8	57	14.04
Eckardt (1996) [7]	1	38	2.63	7	36	19.44
Total	24	322	7.45 (4.83–10.89)	63	327	19.27 (15.13–23.96)

CI, confidence interval; CTFI, chemotherapy-free interval; ORR, objective response rate; SCLC, small cell lung cancer.

Supplementary Table S3 References

1. von Pawel J, Schiller JH, Shepherd FA, Fields SZ, Kleisbauer JP, Chrysson NG, Stewart DJ, Clark PI, Palmer MC, Depierre A, Carmichael J, Krebs JB, Ross G, Lane SR, Gralla R (1999) Topotecan versus cyclophosphamide, doxorubicin, and vincristine for the treatment of recurrent small-cell lung cancer. J Clin Oncol 17 (2):658-667. doi: https://doi.org/10.1200/JCO.1999.17.2.658
2. O'Brien ME, Ciuleanu TE, Tsekov H, Shparyk Y, Cucevia B, Juhasz G, Thatcher N, Ross GA, Dane GC, Crofts T (2006) Phase III trial comparing supportive care alone with supportive care with oral topotecan in patients with relapsed small-cell lung cancer. J Clin Oncol 24 (34):5441-5447. doi:http://doi.org/10.1200/JCO.2006.06.5821
3. von Pawel J, Jotte R, Spigel DR, O'Brien ME, Socinski MA, Mezger J, Steins M, Bosquee L, Bubis J, Nackaerts K, Trigo JM, Clingan P, Schutte W, Lorigan P, Reck M, Domine M, Shepherd FA, Li S, Renschler MF (2014) Randomized phase III trial of amrubicin versus topotecan as second-line treatment for patients with small-cell lung cancer. J Clin Oncol 32 (35):4012-4019. doi:http://doi.org/10.1200/JCO.2013.54.5392
4. Evans TL, Cho BC, Udud K, Fischer JR, Shepherd FA, Martinez P, Ramlau R, Syrigos KN, Shen L, Chadjaa M, Wolf M (2015) Cabazitaxel versus topotecan in patients with small-cell lung cancer with progressive disease during or after first-line platinum-based chemotherapy. J Thorac Oncol 10 (8):1221-1228. doi:http://doi.org/10.1097/JTO.0000000000000588
