# Supplementary material for: Integrated exposure–response analysis of efficacy and safety of lurbinectedin to support the dose regimen in small-cell lung cancer
Source: Cancer Chemother Pharmacol. 2021 Nov 5;89(5):585–94. doi: 10.1007/s00280-021-04366-3 (PMC9054899; doi:10.1007/s00280-021-04366-3)
Supplement: Supplementary file 2 — Supplementary file2 (DOCX 13 KB) [file 280_2021_4366_MOESM2_ESM.docx]

**Supplementary Material:**

Description of bioanalytical method of lurbinectedin

Lurbinectedin plasma concentrations were measured using a validated ultra-performance liquid chromatography tandem mass spectrometry in human K_3_‑EDTA plasma, adding deuterated lurbinectedin (PM01183 –d_4_ or PM040038) as internal standard. The sample extraction was performed using supported liquid extraction. Chromatographic separation was performed using a C_18_, 1.7 µm, 50 x 2.1 mm column and gradient elution with 0.1% ammonium hydroxide in water, acetonitrile. Detection was by triple quadrupole mass spectrometry system with electrospray ionization in positive ion mode; desolvation temperature was established at 400 ºC, capillary voltage at 1 kV, and lurbinectedin and internal standard parent to product transition (*m/z*) were 767.7 to 273.0 and 771.7 to 277.1, respectively. The calibration curves for lurbinectedin displayed good linearity over the concentration range of 0.1 to 50 ng/mL. The intra and inter day precisions ranged from 2.7 to 12.9% and from 5.1 to 10.7%, respectively. Similarly, the within and between day accuracy (bias) ranged from -10 to 12% and -5 to 6%, respectively.
